# Epidural Anesthesia With Low Concentration Ropivacaine and Sufentanil for Percutaneous Transforaminal Endoscopic Discectomy: A Randomized Controlled Trial

**DOI:** 10.3389/fmed.2020.00362

**Published:** 2020-07-31

**Authors:** Lingling Zhang, Tao Chang, Yaru Xu, Qi Jing, Xuan Zhao, Cheng Li

**Affiliations:** Department of Anesthesiology, Shanghai Tenth People's Hospital, Tongji University School of Medicine, Shanghai, China

**Keywords:** epidural anesthesia, herniated disk, local anesthesia, ropivacaine, sufentanil, surgery

## Abstract

**Introduction:** Percutaneous transforaminal endoscopic discectomy is a simple and effective treatment for lumbar intervertebral disc herniation, and local anesthesia is often applied in this kind of surgery in many developing countries, including China. However, many patients still feel excruciating pain under this condition. Epidural anesthesia with low-concentration ropivacaine has no impact on muscle strength, and patients might follow the surgeon well during operation. We hypothesize that epidural anesthesia is feasible for percutaneous transforaminal endoscopic discectomy.

**Methods:** Two hundred patients with disc herniation who underwent percutaneous transforaminal endoscopic discectomy were randomized to receive either epidural anesthesia or local infiltration anesthesia. Primary outcome measures included the pain score, the cooperation degree, and patients' satisfaction. Mean arterial pressure and heart rate were also recorded.

**Results:** Compared with the local anesthesia group, visual analog scale scores, mean arterial pressure, and heart rate were significantly lower in the epidural anesthesia group (*P* < 0.05), and patients' satisfaction was higher. There were no significant differences in the total operation time or blood loss between two groups.

**Conclusions:** Epidural anesthesia with low-concentration ropivacaine and sufentanil is safe and effective for percutaneous transforaminal endoscopic discectomy.

**Clinical Trial Registration:** ClinicalTrials.gov, identifier: ChiCTR-IOR-17011768.

## Introduction

Pain and functional disturbance due to lumbar intervertebral disc herniation have been an important medical and socioeconomic problem (1). It has been confirmed that pain and functional disturbance are associated with herniation of the nucleus pulposus (2, 3), which has a lifetime prevalence exceeding 10% (4). The estimated annual incidence of sciatica in Western countries is five cases per 1,000 adults (5). Although a majority of patients may recover with conservative management, 10–15% of patients still require surgical interventions (6, 7).

With the development of minimally invasive spinal surgery, percutaneous transforaminal endoscopic discectomy (PTED) has become a popular treatment for lumbar intervertebral disc herniation (8) due to its minimal invasiveness, more rapid recovery, and fewer complications (9, 10). Posterolateral access can be utilized for a remedy if the procedure fails (11). There is a specific requirement for the surgery that the surgeon should be timely aware of nerve root symptoms in order to avoid nerve root injury. General anesthesia (GA) may increase the risk for complications because of nerve root anomalies. Intraoperative neurophysiologic monitoring (IONM) can be employed to minimize neurologic injury during surgery (12, 13). However, this procedure has not been widely applied in many developing countries, especially in poor districts, due to the economic concern. Another disadvantage of GA with IONM is that it takes a long time for preoperative preparation. In addition, the real impact of IONM on the neurological outcomes after surgery remains debated although some control studies have been conducted (14). It has been indicated that local anesthesia (LA) is preferable to GA during endoscopic discectomy (15). LA or LA plus intravenous anesthesia are now commonly used (16–19), but the patients' satisfaction about analgesia is poor, and only 50% of patients are satisfied with intra-operative analgesia (17). Epidural anesthesia (EA) with ropivacaine and sufentanil has been used in an effort to facilitate painless childbirth, and it has proven to be safe and effective (20, 21). However, little is known about EA with ropivacaine and sufentanil for PTED. This study assesses the effects of EA with low-concentration ropivacaine and sufentanil on pain relief in patients receiving PTED and analyzes the degree of intraoperative cooperation and patients' satisfaction.

## Methods

### Patients

This study was approved by the Ethical Committee of the Shanghai Tenth People's Hospital, Shanghai, China (No. SHSY-IEC-KY-4.0/17-7/01). Clinical trial number and registry URL are ChiCTR-IOR-17011768 and http://www.chictr.org.cn, respectively. Inclusion criteria were as follows: (1) patients were aged 18–60 years, (2) the American Society of Anesthesiologists (ASA) physical status was I or II, and (3) patients received PTED at the L5-S1 between November 2015 and October 2016. Exclusion criteria were as follows: (1) patients received anticoagulant therapy before surgery or had coagulation dysfunction, (2) patients had systemic or local infection or were allergic to drugs used in this study, (3) patients had severe cardiopulmonary dysfunction, or (4) patients had abnormal mental status. After the written informed consent was obtained, patients were randomized to receive EA (EA group) or local infiltration anesthesia (LA group). Estimations were performed by another anesthesiologist who was blind to the grouping. All patients were diagnosed with protrusion of the lumbar intervertebral disc at L5-S1 with unilateral symptoms.

### Procedures

A venous access was established before surgery, and electrocardiogram, pulse oximetry, non-invasive blood pressure (BP), and heart rate (HR) were monitored during the surgery. Patients in the EA group were positioned on the right side, and an epidural catheter was introduced between L2–3 level via a midline approach using a 16G needle. To identify the epidural space, a loss of resistance to normal saline technique was used. The catheter (single-use puncture set for local anesthesia, TuoRen, XinXiang, China) was advanced 3 cm into the epidural space in a cephalad direction. The level was verified by C-arm fluoroscopy before puncture. If the epidural insertion failed, other anesthesia was administered and patients were excluded from this study. Two percent lidocaine (Lidocaine Hydrochloride Injection, HuaLu, Liaocheng, China) (3 ml) was injected for a test, and then, a bolus of 10 mL epidural analgesic with 0.125% ropivacaine (Ropivacaine Hydrochloride Injection, HengRui, Lianyungang, China) in combination with 0.2 μg/mL sufentanil (Sufentanil Citrate Injection, RenFu, Yichang, China) was administered. Thirty minutes after the bolus dose, bilateral symmetric sensory was blocked at the Th 8–10 level with lower limbs movable freely. The epidural catheter was removed at the end of the operation. The vital signs were monitored for 6 h after surgery. Patients in the LA group received local infiltration anesthesia with 13 ml of 1% lidocaine. Patients in both groups were awake and had no sedation, and no rescue analgesia was used in either group. Visual analog scale (VAS) scores, degree of intraoperative cooperation, and patients' satisfaction were recorded as the primary outcomes, and mean arterial pressure (MAP) and HR were recorded as the secondary outcomes at following time points: T1, the guide needle broke the skin; T2, the wire guide entered the fiber ring; T3, windowing of annulus fibrosus; T4, resection of the nucleus pulposus; T5, radio frequency ablation; T6, thermocoagulation. Patients were asked to score their satisfaction with the anesthesia with a numerical scale: 8–10, “very satisfied”; 4–7, “satisfied”; <4, “not satisfied.” Surgical onset time, total operation time, and blood loss were also recorded. When the degree of intraoperative cooperation was assessed, no unplanned body movement was defined as very cooperative, infrequent unplanned body movement as cooperative, and unplanned body movement disturbing the operation as not cooperative.

### Statistical Analysis

Sample size analysis: an intergroup difference of two points in pain score was considered to be clinically significant in our study because the significant pain was a 5 score on preliminary observations in this study population. VAS <3 indicated that the pain was slight and tolerable. Accordingly, in our prior assessment, 82 patients were required with 80% power at a two-tailed significance level of 0.05 to detect a reduction of two points in the pain score. One hundred patients were, therefore, enrolled in each group. Statistical analysis was performed with SPSS version 20.0. Qualitative variables, such as patients' satisfaction and degree of intraoperative cooperation, are expressed as frequencies and percentages and quantitative variables, such as VAS, HR, MAP, total operation time, and blood loss, as means ± standard deviations (SD). Comparisons of quantitative variables were done using analysis of variance (ANOVA). The Pearson chi-square test was used for comparisons of patients' satisfaction and degree of intraoperative cooperation. A value of *P* < 0.05 was considered statistically significant.

## Results

### General Data

**Figure 1:**
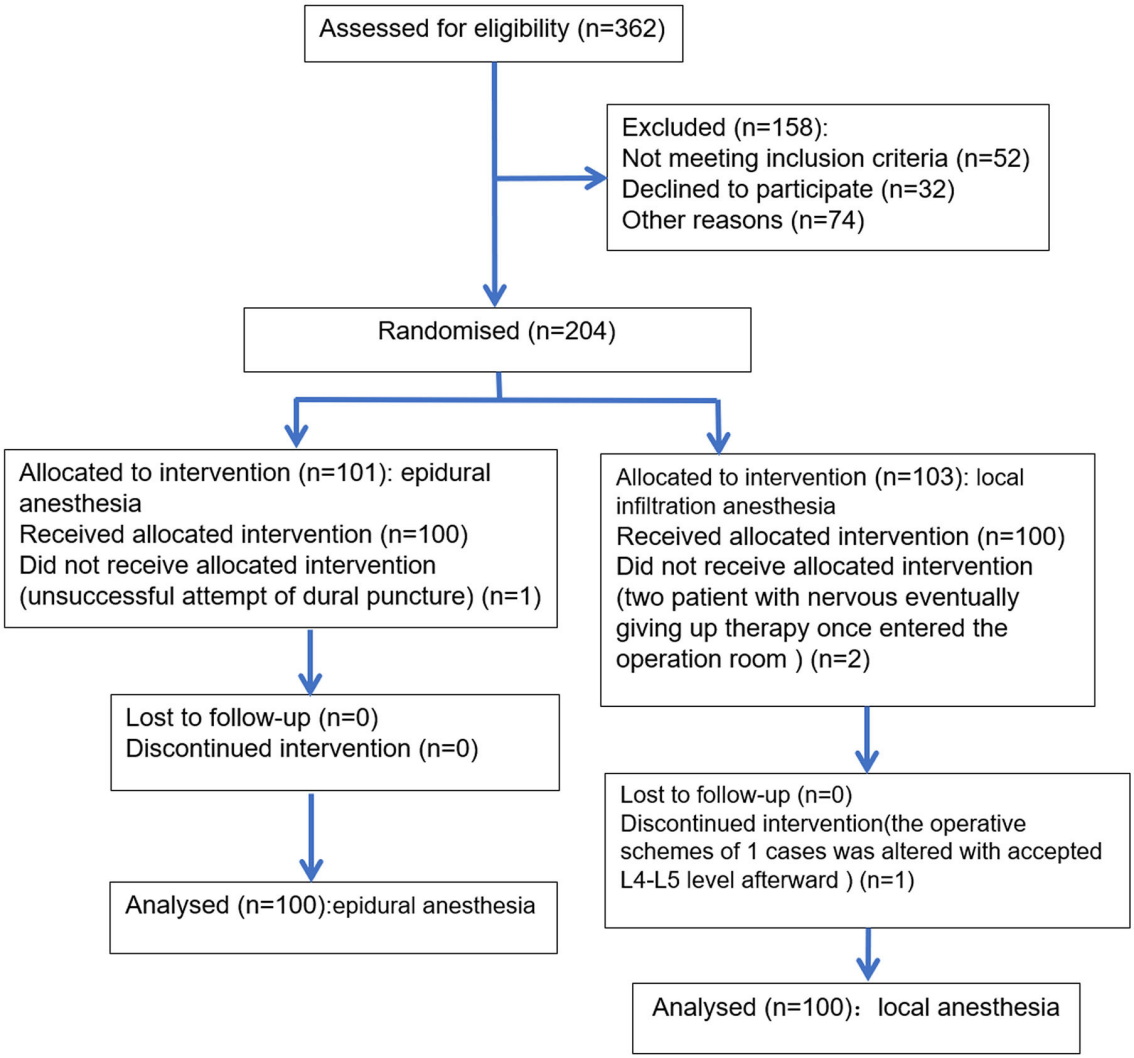
Flowchart of selection process.

Two hundred patients were enrolled into this study between November 2015 and October 2017 with 100 in the LA group and 100 in the EA group. Figure 1 shows the flow diagram. There were no significant differences in the demographics or preoperative clinical features between the LA and EA groups, indicating it was comparable between groups (Table 1). All patients were operated on by the same team. The sensory level of anesthesia in the EA group was higher than T10 and lower than T6. The major muscle force of both lower limbs was preserved above three grades.

**Table 1:** Demographic characteristics of patients in two groups.

**Item**	**EA group**	**LA group**
Number	100	100
Female (*n*)	51	47
Male (*n*)	49	53
Age (Years)	40.01 ± 11.18	39.68 ± 11.56
ASA classification		
I	29	33
II	71	67
Visual analog scale (preoperative back pain)	4.17 ± 1.31	3.98 ± 1.24
Visual analog scale (preoperative leg pain)	6.27 ± 1.08	6.41 ± 1.31
Oswestry disability index	46.73 ± 10.35	48.22 ± 14.53

*Data are present as mean ± SD. ASA, American Society of Anesthesiologists; EA, Epidural anesthesia; LA, Local anesthesia*.

### Primary Endpoints

**Figure 2:**
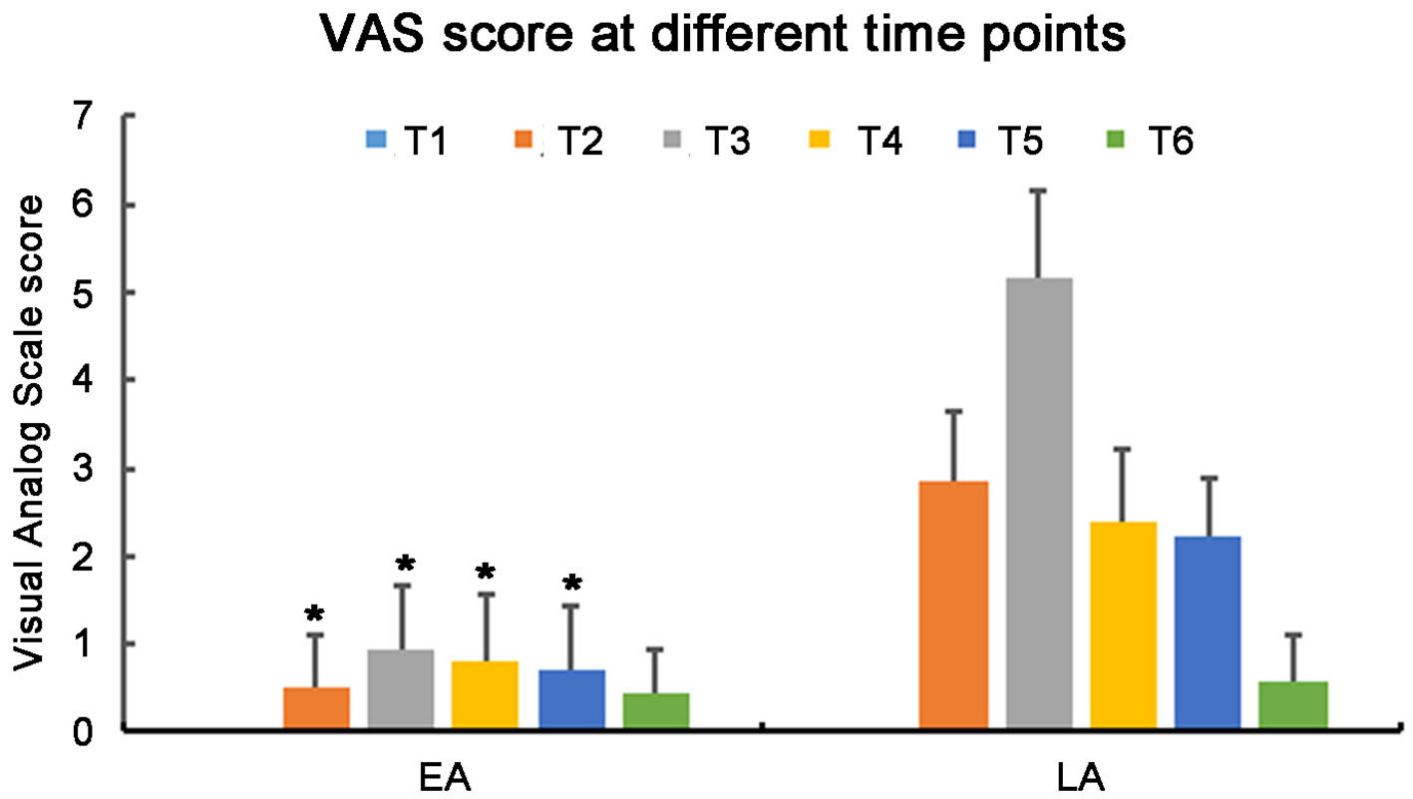
Visual analog scale score in the EA group and LA group at different time points. EA, Epidural anesthesia; LA, Local anesthesia. *p < 0.001.

At T2–T5, the VAS scores were significantly lower in the EA group than in the LA group (T2–T5: *P* < 0.001). Analgesia was effective during the surgery in the EA group with VAS scores <4 at all-time points. There were no significant differences in the VAS scores between two groups at T1 and T6 (Figure 2, T6: *P* = 0.253). All patients in both groups could timely tell the paresthesia and the site of its radiations when the surgeon touched the spinal nerve, but the patients' cooperation in the LA group was poorer than in the EA group (83 vs. 98%, *P* = 0.001). Postoperative patients' satisfaction was 72 and 100% in the LA and EA groups, respectively (*P* < 0.001). All patients in the EA group were satisfied with the anesthesia and reported that they would accept the same procedure again if a second surgery was required, but many patients in the LA group reported that it was hard to tolerate the pain during the surgery.

### Secondary Endpoints

MAP and HR increased in the LA group at T2–T5 (HR: T2: *P* = 0.029; T3: *P* < 0.001; T4: *P* = 0.034; T5: *P* = 0.036; MAP: T2–T5: *P* < 0.001), and there were no significant changes in the HR in either group at T1 and T6 (T1: *P* = 0.511; T6: *P* = 0.739, Table 2). MAP was markedly lower in the EA group at T6 (*P* < 0.001). Surgical onset time was longer in the EA group than in the LA group (*P* < 0.001), but there was no significant difference in the total operation time between the two groups (*P* = 0.410), and the intraoperative blood loss was also comparable between the two groups (*P* = 0.310, Table 3). None in either group experienced serious adverse events or complications.

**Table 2:** Heart rate and mean arterial pressure in two groups at different time points.

**Parameters**	**Group**	**T1**	**T2**	**T3**	**T4**	**T5**	**T6**
Heart rate (bpm)	EA	80.04 ± 7.44	78.44 ± 7.05^*^	79.82 ± 6.95^*^	77.34 ± 7.53^*^	78.48 ± 7.19^*^	78.22 ± 7.29
	LA	79.08 ± 7.11	81.46 ± 6.57	88.30 ± 6.03	80.42 ± 6.75	81.32 ± 6.10	77.74 ± 7.08
MAP (mmHg)	EA	99.20 ± 14.03	95.58 ± 4.89^*^	95.44 ± 4.54^*^	94.64 ± 3.87^*^	94.40 ± 3.89^*^	94.48 ± 3.73^*^
	LA	100.52 ± 7.05	102.86 ± 6.90	105.36 ± 5.68	104.68 ± 5.23	101.24 ± 3.70	100.76 ± 3.42

Data are present as mean ± SD. bpm, beat per minute; MAP, Mean arterial pressure; EA, Epidural anesthesia; LA, Local anesthesia;

* *P < 0.05*.

**Table 3:** The surgical onset time, total operation time, and bleeding volume in the EA and LA groups.

**Item**	**EA**	**LA**
Surgery onset time (mine)	19.78 ± 3.34^*^	9.82 ± 2.82
Total operation time (mine)	143.56 ± 15.53	147.18 ± 19.67
Bleeding volume (mL)	115.96 ± 11.17	118.00 ± 13.39

Data are present as mean ± SD. EA, Epidural anesthesia; LA, Local anesthesia;

* *P < 0.05*.

## Discussion

Lower back pain affects 80% of the general population, has been one of the most common complaints, and has a tremendous impact on society and the economy (22, 23). Current treatments for lower back pain include education, self-care, medications, physical therapy, epidural injection, and microdiscectomy for those who are poorly responsive to conservative treatments (2, 7). Discectomy is the most frequently used spinal surgery, and the traditional open approach has been replaced by endoscopic discectomy, which has become the gold standard treatment (11). Endoscopic discectomy has the following advantages: (1) reduced traumatization, hospital stay, and postsurgical morbidity; (2) direct visualization of the intervertebral space; (3) reduced blood loss; and (4) a high degree of patient satisfaction (24). PTED has become a popular minimally invasive surgery for the lumbar intervertebral disc herniation as a main cause of lower back pain (17).

To prevent over-irritation of the dural sac, nerve root, and excessive neural manipulation or damage, GA without IONM is infeasible, and most surgeons often complete the surgery with LA in poverty-stricken areas, but as a result, patients usually feel discomfort during the surgery (17). This discomfort may result in hemodynamic changes and increased cardio-cerebral vascular incidents during the operation (25).

Patients with preexisting spinal canal pathology are not often considered candidates for neuraxial blockade. Available studies have indicated that preexisting spinal canal pathology may be a significant contributor to the neurologic complications after neuraxial block (26). The mechanisms of injury may include ischemia, mechanical trauma, local anesthetic toxicity, and others (27). However, the available studies usually have a small sample size, and few prospective, randomized controlled studies with large sample size have been conducted. Spinal anesthesia has been safely used for lumbar spine surgery (28), and the reported spinal anesthesia is superior to GA without increasing adverse effects (29). Recently, increasing evidence has suggested that the risks commonly associated with neuraxial anesthesia and analgesia in patients with preexisting CNS disorders may not be as frequent as once thought and that neuraxial blockade should not be considered an absolute contraindication within this patient population (30).

It is reported that epidural anesthesia is a useful option for anesthesia in patients undergoing percutaneous endoscopic lumbar discectomy, but the safety and effectiveness have never been assessed comprehensively (31). A low-concentration ropivacaine combined with sufentanil has proven to be effective and safe to facilitate painless childbirth (20, 21). In the present study, this approach was successfully applied for PTED. Our results show that this approach could provide adequate pain relief and also facilitate patients to cooperate with surgeons. Ropivacaine, one of the newer local anesthetics, may result in lower extremity motor block than bupivacaine after epidural administration (32) and has advantages in painless childbirth and PTED. The motor block can be minimized by reducing the concentration of ropivacaine with the addition of opioids (33). It is also worth emphasizing that epidural injection is an option for the treatment of lumbar pain (34) although some researchers have suggested that the associated benefits are small and not sustained (35). EA not only increases the degree of satisfaction, but also improves the effectiveness of the operation.

In the present study, patients had increased pain at T3, especially in the LA group, and additional analgesic was often needed at this time point. In our study, there were no significant differences in the VAS scores between two groups at T6. It has been reported that thermocoagulation can cause sharp local temperature increase in the intervertebral discs and that thermal energy may destroy pain receptors in the intervertebral discs and pain-sensitive nerve endings in the external anulus, resulting in the improvement of surgical pain (36).

GA with IONM is a good choice, but it needs special medical equipment and has higher costs; LA is the most commonly used anesthesia method due to its convenience, high security, and inexpensiveness, but it might be less effective. As an alternative, EA with low-concentration ropivacaine is safe, effective, and especially applicable in most developing countries. Some awake patients with EA may feel uncomfortable in the prone position. Maybe EA with sedation is a choice.

One of the limitations in the present study was that there was no control group of GA with INOM, the most commonly used anesthetic technique in the developed countries. In addition, we only recorded data during surgery and did not investigate the pain scores, muscle strength, spontaneous urination, or surgical outcomes after surgery.

## Conclusions

In summary, our results show that both LA and EA are effective for PTED. However, in our opinion, epidural ropivacaine with sufentanil may be a better choice for PTED because it provides adequate pain relief and also facilitates patients to cooperate with surgeons.

## Data Availability Statement

The raw data supporting the conclusions of this article will be made available by the authors, without undue reservation.

## Ethics Statement

The studies involving human participants were reviewed and approved by Ethical approval for this study (No. SHSY-IEC-KY-4.0/17-7/01) was provided by the Ethical Committee of Shanghai Tenth People's Hospital, Shanghai, China. Clinical trial number and registry URL are ChiCTR-IOR-17011768 and http://www.chictr.org.cn. The patients/participants provided their written informed consent to participate in this study.

## Author Contributions

LZ, TC, XZ, and CL: conception and design. CL: administrative support. LZ, TC, YX, QJ, XZ, and CL: provision of study materials or patients and collection and assembly of data. LZ and TC: data analysis and interpretation. All authors: manuscript writing and final approval of manuscript.

## Conflict of Interest

The authors declare that the research was conducted in the absence of any commercial or financial relationships that could be construed as a potential conflict of interest.
